# Sensory dysfunction in SMA type 2 and 3 - adaptive mechanism or concomitant target of damage?

**DOI:** 10.1186/s13023-024-03339-y

**Published:** 2024-09-03

**Authors:** Magdalena Koszewicz, Jakub Ubysz, Edyta Dziadkowiak, Malgorzata Wieczorek, Slawomir Budrewicz

**Affiliations:** 1 Clinical Neurophysiology Laboratory, University Centre of Neurology and Neurosurgery, Faculty of Medicine, Wroclaw Medical University, Borowska 213, Wroclaw, 50-556 Poland; 2 Clinical Department of Neurology, University Centre of Neurology and Neurosurgery, Faculty of Medicine, Wroclaw Medical University, Borowska 213, Wroclaw, 50-556 Poland; 3 Faculty of Earth Sciences and Environmental Management, University of Wroclaw, Uniwersytecki Square 1, Wroclaw, 50-137 Poland

**Keywords:** Spinal muscular atrophy, Sensory nerve action potential, Sensory conduction velocity, Quantitative sensory testing, Hammersmith functional motor scale – expanded

## Abstract

**Background:**

The motor neuron survival protein performs numerous cellular functions; hence, spinal muscular atrophy (SMA) is considered to be a multi-organ disease with possible sensory system damage. The controversy surrounding the presence of sensory disturbances, prompted us to conduct standard electrophysiological studies and assess the sensory thresholds for different modalities in adults with SMA types 2 and 3. The study group consisted of 44 adult SMA patients (types 2 and 3). All patients underwent neurological examination using the Hammersmith Functional Motor Scale – Expanded (HFMSE). Standard sensory electrophysiological studies in the ulnar nerve and the estimation of vibratory, temperature, and warm- and cold-induced pain thresholds with temperature dispersion assessment were performed using quantitative sensory testing (QST).

**Results:**

The most repeatable result was the high amplitude of the sensory nerve action potentials (SNAP) in SMA patients compared to controls. This was higher in type 2 patients compared to type 3a and 3b patients and patients with low HFSME scores. Patients with SMA, especially type 3b presented a longer sensory latency and slower conduction velocity than did controls. Cold pain threshold was higher and warm dispersion larger in SMA. The vibratory limit was higher in patients with high HFSME scores.

**Conclusions:**

A high SNAP amplitude suggests sensory fibre hyperactivity, which may be based on overactivation of metabolic pathways as an adaptive mechanism in response to SMN protein deficiency with additionally coexisting small C- and A-delta fibre damage. SMA patients seem to have a concomitant, mild demyelinating process present at the early SMA stage.

## Background

Spinal muscular atrophies are a group of several heterogenic progressive neuromuscular disorders characterized by loss of spinal cord and brainstem alpha motoneurons that leads to skeletal muscle weakness and atrophy. Among patients with SMA, there are different forms of the disease, with similar motor symptoms but differing in their severity, molecular biology, and inheritance pattern. The most frequent disease in this group is proximal spinal muscular atrophy (SMA), which accounts for approximately 95% of all spinal muscular atrophies [1–3]. SMA is caused by mutations in the survival motor neuron 1 (SMN1) gene, localized on chromosome 5q13. This leads to deficiency in the SMN protein, which is essential for the survival and function of lower motor neurons [1, 4].

SMA seems not to be an isolated alpha motoneuron disorder. Symptoms from other organs are also present, especially in clinically advanced forms of disease; currently, SMA is regarded as a systemic disease. Abnormalities have also been found in various structures of the central and peripheral nervous system in addition to motoneurons [5–8]. The contraction of the muscle results from the correct activity of alpha motorneurons which depends on regulatory processes and feedback with the sensory system. The sensory–motor circuit is subject to regulatory processes and complex feedbacks which are influenced by central nervous system and peripheral sensory inputs. In SMA, the circuit appears to be defective in its various parts [6, 9–12]. The literature based on human and animal studies and case reports shows damage at the level of sensory nerves and the whole sensory system [6, 10, 11, 13].

The controversy surrounding the presence of sensory disturbances in SMA, prompted us to undertake research on this topic. In our study, we analyzed the sensory function of the ulnar nerve using the standard sensory conduction tests together with temperature, pain, and vibratory threshold estimations obtained by quantitative sensory testing (QST) in order to find any peripheral sensory abnormalities in patients with SMA types 2 and 3 and having various degrees of disability.

Methods.

The study was approved by the Ethics Committee of Wroclaw Medical University in Poland, and conducted in accordance with the principles of good clinical practice (GCP). We obtained informed consent forms from all participants in the study.

44 adult patients (19 female, 25 male) with SMA and 32 healthy volunteers (19 female, 13 male) were included in the study. SMA was confirmed by genetic testing, and the number of gene copies was assessed. We analyzed patients with SMA types 2 and 3, and we distinguished the subgroups 3a and 3b on the basis of age at symptom onset, i.e., before and after 3 years of age, respectively [4]. Body mass index (BMI) was assessed in our patients. We did not exclude patients with comorbidities due to their low degree of severity, even though some of these comorbidities could influence the results. Diabetes mellitus treated orally was present in three patients; a further three patients had supplemented hypothyroidism; one had pituitary microadenoma hormonally inactive; and one patient had psoriasis. In our study group, more than 70% of patients had other medical conditions (a separate article on comorbidity in SMA is currently under review in another journal). Therefore, excluding these patients from the study would have prevented any statistical analysis. Standard electrophysiological tests were performed to exclude obvious polyneuropathy in these patients, but their results were not analyzed in this article. The study group was the same as previously described elsewhere [14].

All the scheduled tests were performed before the patients started the treatment. In all patients, we performed a neurological examination with a rating on the Hammersmith Functional Motor Scale – Expanded (HFMSE) [15], and Visual Analogue Scale (VAS) for the assessment of chronic pain [16]. Electrophysiological evaluations with quantitative sensory testing (QST) were performed in all patients and healthy volunteers.

The electrophysiological studies were conducted using the following device: Viking Quest version 10.0 (Viasys Healthcare Inc., Consohocken, Pennsylvania, USA) with device attachments: Thermal Sensory Analyzer II 2001 (TSA II) and VSA – 3000 Vibratory Sensory Analyzer (Medoc, Israel).

Sensory conduction tests were performed in the ulnar nerve according to standard procedures using the antidromic technique [17, 18]. Ring recording electrodes were used, and sensory nerve action potentials (SNAP) were obtained from the fifth digit. Electrical stimulation was performed at the wrist, while maintaining a standard distance from the recording electrode equal to 12 cm. The standard room temperature was 21–23ºC; hand temperature was not lower than 32ºC. Distal latency (in milliseconds – ms), amplitude (in microvolts – uV), and conduction velocity (in meters per second – m/s) were assessed.

QST allows estimation of the sensation and pain thresholds for cold and warm temperatures and additionally uses a special device module – vibration threshold. The following thresholds were estimated using limit methods: cold sensation (CS), warm sensation (WS), cold pain (CP), heat pain (HP), and vibration sensation (VS). We also analyzed the temperature differences between CS and CP, and WS and HP (the dispersion of the temperature). Thermal stimuli were produced by a thermode (Peltier modules). The thermode active area is 30 × 30 mm, temperature range 0–50.5 °C. The thermode was attached to the skin of the palm on the hypothenar region. The temperature changed by 1 °C/s during temperature threshold estimation and 2 °C/s when the pain threshold was evaluated. The basic temperature (adaptation temperature) was 32 °C. When the patients felt cold, warm, or pain, the stimulation was stopped by pressing a button. This was a subjective part of the study. The procedures were repeated four times for temperature and three times for pain threshold estimation. The thresholds were calculated as the average values in degrees Celsius [19–21]. A vibratory sensation analyzer was used to measure thresholds for vibratory stimuli. Patients put their little finger on a vibrating button with a stimulating area equal to 1.22 cm^2^. The vibratory stimulation rate was 100 Hz, the amplitude ranged from 0 to 130 microns (µ), and the amplitude changed with a rate of 0.3 microns per second (µ/s). When the patients felt vibration, the stimulation was stopped by pressing a button. The vibration threshold was calculated as an average value from six repetitions [19, 20].

STATISTICA 13.0 software was used for statistical analysis. The number of cases (N), mean (X), median (M), and standard deviations (SD) of continuous parameters were estimated. The Shapiro–Wilk test was used to assess the normality of the distribution. Depending on the distribution of the variables, the Student’s t-test and Mann–Whitney U-test were used for comparative analysis of mean values. ANOVA was used to assess variance; in the absence of a normal distribution in the subgroups, the Kruskal–Wallis test was performed instead of ANOVA. When the distribution of variables was not normal, Spearman’s rank correlation coefficient was calculated. A ratio test was performed to assess whether the control group matched the patient group in terms of gender and age composition. All tests were performed at a significance level of α = 0.05, with Bonferroni correction.

## Results

The patient group consisted of 19 females and 25 males, with a mean age of 36.09 ± 10.98 years. The control group, which consisted of 19 females and 13 males, had a mean age of 44.31 ± 13.03 years. The groups did not differ significantly in terms of gender (*p* = 0.16) but differed in terms of age. Age differences between the groups of patients with different types of SMA did not reach statistical significance (*p* = 0.0728). 43 patients were right-handed; one was left-handed. A description of the patient group in terms of type of SMA, age, BMI, gene copies, HFSME scores is presented in Table 1.

**Table 1:** Demographics of the SMA patient group

SMA type	Patients (*n*)	Sex F/M	Age (mean ± SD in years)	BMI	Gene copies (*n*)	Symptoms onset (mean ± SD in years)	HFSME (points ± SD)
2	9	7/2	30,11 ± 8,02	18,05 ± 4,53	2 − 1 3 − 7 4 − 1	1.7 ± 0.50	2.56 ± 2.07
3a	21	7/14	35,38 ± 11,75	23,77 ± 5,49	2 − 0 3 – 12 4 – 9	2.33 ± 0.80	16.00 ± 15.51
3b	14	5/9	41,00 ± 9,77	23,38 ± 3,80	2 − 0 3 – 8 4 – 6	10.79 ± 3.68	33.57 ± 17.43

SMA – spinal muscular atrophy, n – number of subjects, SD – standard deviation, F – female, M – male, HFSME – the Hammersmith Functional Motor Scale – Expanded

Overweight/obese (BMI > 25) was seen in 9 (20,5%) SMA patients, underweight (BMI < 18.5) in next 9 (20,5%). BMI differed between SMA patients and controls (22.20 ± 5.19 and 24.44 ± 2.22, respectively, *p* = 0.017), with a BMI range of 10.8 to 33.1 in SMA patients and 21.1 to 33.3 in controls. In type 2 SMA, mean BMI was significantly lower than in types 3a and 3b (*p* = 0.006 and *p* = 0.009, respectively) (Table 1). On clinical examination, none of the patients presented sensory abnormalities suggestive of polyneuropathy. Some of them, especially those with advanced scoliosis, complained of pain in the spine region and lower limbs. In none of our patients did the VAS score exceed 5 for chronic pain.

Standard sensory conduction study and QST in the patient and control groups.

Unexpected results were determined concerning SNAP amplitude. This was statistically higher in patients with SMA (*p* < 00001) (Table 2). The range of SNAP amplitudes in the patient group was 19.1 to 138.9.uV, while in the control group it ranged from 6 to 81uV. Only one patient, a 26-year-old male with SMA type 3b (disease onset at 12 years of age and an HFSME score of 30), failed to achieve a sensory response in the examined nerve. In the other examined nerves in this patient (median nerve, sural nerve on the left side) SNAP responses were present, so the lack of sensory response in the ulnar nerve was considered to be an incidental finding. SNAP latency was statistically longer (*p* = 0.019), and conduction velocity was statistically slower (*p* = 0.0007) in the SMA group than in the control group (Table 2).

**Table 2:** Standard sensory conduction test and QST in the SMA and control groups

Ulnar nerve	Study goup *n* = 44		Control group *n* = 32		*p*-value
	mean	SD	mean	SD	
L (ms)	2.26	0.37	2.10	0.32	0.019
A (uV)	67.96	27.23	35.34	17.25	< 0.00001
CV (m/s)	49.13	7.02	54.66	6.19	0.0007
CS (°C)	29.32	2.39	29.39	1.31	0.343
WS(°C)	34.42	1.04	34.33	1.04	0.598
CP (°C)	20.25	5.01	23.43	3.22	0.004
HP (°C)	41.37	4.57	40.30	3.96	0.354
HP-WS(°C)	9.05	4.13	5.56	2.79	0.001
CS-CP (°C)	7.14	4.47	6.55	4.11	0.563
VBL (u)	1.16	0.82	1.30	1.29	0.249

SMA – spinal muscular atrophy, QST – quantitative sensory testing, L – latency, A – amplitude, CV – conduction velocity, CS – cold sensation, WS – warm sensation, CP – cold pain, HP – heat pain, HP-WS – warm dispersion, CS-CP – cold dispersion, VBL – vibratory limits, ms – milliseconds, uV – microvolts, m/s – meters per second, s – seconds, u – microns, °C – Celsius degree, n – number of subjects

In QST assessment, CP threshold was significantly higher in SMA than in controls, and HP-WS was significantly larger in the SMA group. HP threshold, and cold temperature dispersion were higher and greater, respectively, in SMA but without statistical significance (Table 2).

Standard sensory conduction study and QST in relation to the type of SMA.

**Fig. 1:**
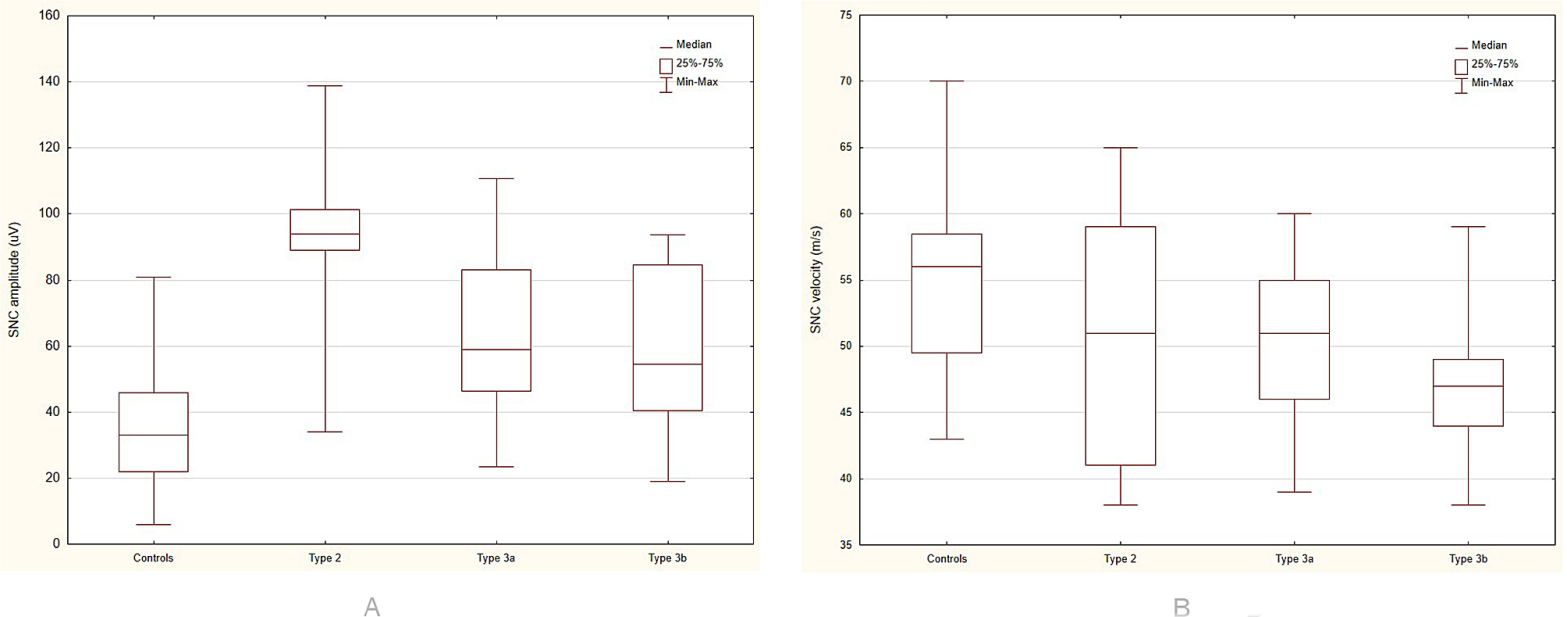
**A.** Box plots for sensory mean amplitudes in the ulnar nerve in patients with SMA types 2, 3a, and 3b and controls. **B.** Box plots for sensory mean conduction velocity in the ulnar nerve in patients with SMA types 2, 3a, and 3b and controls. SNC – sensory nerve conduction, uV – microvolts, m/s- meters per second

A comparison of the parameters of a standard sensory conduction study in different types of SMA revealed differences only in SNAP amplitudes between groups (*p* = 0.0137). Significantly, the highest amplitude was observed in SMA type 2, while this was lower in type 3a and the lowest one in SMA type 3b; this was confirmed using multiple comparisons (Fig. 1A). SNAP latency (*p* = 0.1380) and conduction velocity (*p* = 0.5704) did not differ significantly between the SMA types. Conduction velocity tended to be slower in SMA type 3b than type 2 and 3a (SMA type 3b − 47.54 ± 6.27, type 3a- 49.51 ± 6.02, type 2–50.56 ± 8.11) (Fig. 1B). Exceptions were patients with types 3b SMA, in whom all parameters (amplitude, latency, and conduction velocity) differed significantly from the control group. Amplitude was significantly higher in the patient group (*p* = 0.0004), latency significantly longer (*p* = 0.011), and sensory conduction velocity was significantly slower (*p* = 0.001) (Table 3). QST parameters did not differ significantly between groups. A comparison of CP values in the patients with SMA3b and controls revealed significantly lower cold pain thresholds in the patient group and, although not statistically significant, the temperatures for CP values were the lowest in patients with SMA type 3b and the highest in patients with SMA type 2 (22.21 ± 3.40 in type 2, 21.10 ± 3.61 in type 3a, and 17.72 ± 6.72 °C in type 3b). Similar to the patient group as a whole, patients with SMA type 3b had significantly larger difference in HP-WS than in the control group. CP-CS was larger, and HP was higher in SMA type 3b than in the control group but without statistically important differences (Table 3).

**Table 3:** Standard sensory conduction test and QST in patients with SMA type 3b and control group

Ulnar nerve	SMA type 3b *n* = 14		Control group *n* = 32		*p*-value
	Mean	SD	Mean	SD	
L (ms)	2.32	0.27	2.10	0.32	0.011
A (uV)	59.50	23.29	35.34	17.25	0.0004
CV (m/s)	47.54	6.2665	54.66	6.19	0.001
CS (°C)	28.79	3.30	29.39	1.31	0.971
WS(°C)	34.73	1.46	34.33	1.04	0.389
CP (°C)	17.72	6.72	23.43	3.22	0.0003
HP (°C)	42.54	5.06	40.30	3.96	0.124
P-WS(°C)	11.06	5.18	5.56	2.79	0.001
CS-CP (°C)	8.39	4.96	6.55	4.11	0.198
VBL (u)	1.40	1.29	1.30	1.29	0.183

SMA – spinal muscular atrophy, QST – quantitative sensory testing, L – latency, A – amplitude, CV – conduction velocity, CS – cold sensation, WS – warm sensation, CP – cold pain, HP – heat pain, HP-WS – warm dispersion, CS-CP – cold dispersion, VBL – vibratory limits, ms – milliseconds, uV – microvolts, m/s – meters per second, s – seconds, u – microns, °C – Celsius degree, n – number of subjects

Standard sensory conduction study and QST in relation to the number of gene copies.

We excluded from the analysis one patient who had two copies of the SMN2 gene; we only compared patients with three and four copies. SNAP latency, conduction velocity and amplitude, and QST parameters did not differ between SMA patients with three and four gene copies.

Standard sensory conduction study and QST in relation to HFSME score.

The study group was divided into two subgroups according to the HFSME score, i.e., a subgroup with low scores equal to or less than 10 points and a subgroup with high scores above 10 points.

Patients with low HFSME scores had significantly higher SNAP amplitudes than patients with better results on the HFSME scale (Table 4). They did not differ in terms of SNAP latency and sensory conduction velocity in the basic evaluation. Following the Kruskal-Wallis test for all SCN variables, statistically significant differences were shown between the SMA groups with high and low HFSME scores and the control group. SNAP latency was longer, and conduction velocity was slower in both SMA groups in comparison to the controls (Table 5).

**Table 4:** The comparison of standard sensory conduction test and QST between SMA patients with low and high HFSME score

Ulnar nerve	HFSME < = 10 *n* = 23		HFSME > 10 *n* = 21		*p*-value
	Mean	SD	Mean	SD	
L (ms)	2.19	0.42	2.33	0.29	0.374
A (uV)	77.99	27.64	56.44	22.20	0.008
CV (m/s)	49.17	7.55	49.09	6.55	0.968
CS (°C)	29.70	0.97	28.90	3.30	0.972
WS(°C)	34.26	0.66	34.60	1.33	0.814
CP (°C)	21.73	3.67	18.63	5.83	0.051
HP (°C)	41.16	4.95	41.60	4.22	0.581
HP-WS(°C)	7.94	3.82	10.27	4.20	0.061
CS-CP (°C)	6.90	4.87	7.40	4.08	0.718
VBL (u)	0.91	0.36	1.44	1.07	0.006

SMA – spinal muscular atrophy, QST – quantitative sensory testing, HFSME – Hammersmith Functional Motor Scale – Expanded, L – latency, A – amplitude, CV – conduction velocity, CS – cold sensation, WS – warm sensation, CP – cold pain, HP – heat pain, HP-WS – warm dispersion, CS-CP – cold dispersion, VBL – vibratory limits, ms – milliseconds, uV – microvolts, m/s – meters per second, s – seconds, u – microns, °C – Celsius degree, n – number of subjects

**Table 5:** Kruskal-Wallis test for SNC variables (latency, amplitude, conduction velocity) in groups of patients with low and high HFSME scores and controls

Ulnar nerve	HFSME < = 10	HFSME > 10	Controls	*p*-value
	Mean	Mean	Mean	
SNC L (ms)	2.19	2.33	2.10	0.0357
SNC A (uV)	77.99	56.44	35.34	< 0.0001
SNC CV (m/s)	49.17	49.09	54.66	0.006

SMA – spinal muscular atrophy, HFSME – Hammersmith Functional Motor Scale – Expanded, L – latency, A – amplitude, CV – conduction velocity ms – milliseconds, uV – microvolts, m/s – meters per second

QST parameters were comparable between the groups, while vibratory limits were significantly higher in the group with better HFSME scores (Table 4).

## Discussion

The choice of the ulnar nerve for our study was dictated by the very large anatomical changes in the lower limbs with irreducible contractures in some SMA patients, as well as the presence of clinical features of carpal tunnel syndrome in other patients in the study group. The distal upper limbs are usually less affected than the lower limbs due to muscle atrophy and osteoarticular deformities in SMA 5q.

The most repeatable result of our study were the changes in the amplitude values of the sensory potentials. The amplitudes in the analyzed individual patient groups differed between those groups and in relation to the controls. The SNAP amplitudes were significantly higher in the study group compared to the control group, higher in the more severe forms of SMA, i.e., in type 2 compared to 3a and 3b, respectively. Amplitudes were also significantly higher in the group of patients with a more severe degree of disability as expressed by the HFSME scale. Maintaining an appropriate skin temperature, i.e. always above 32 degrees, seems to exclude the influence of this factor on the amplitude value. Assessing the impact of overweight and underweight based on BMI in SMA patients is difficult due to the heterogeneity of this group of patients; among others, some patients show marked overweight coexisting with muscular atrophy.

Large SNAP amplitudes have been previously described in spinal cord disease. Pullman et al. [22] described the occurrence of high SNAP amplitude in patients with myelopathy without identifying the possible cause. High SNAP amplitude seems to be proportional to the number of active sodium channels per unit of membrane area. In the case of spinal cord damage, axonal transport can be redirected to healthy axons of cells. This phenomenon may result in an increased density of sodium channels and other transported substances in the peripheral, healthy parts of neurons. It is probable that metabolic overactivity is an adaptive mechanism in response to damage [22, 23].

Tripton et al. [23] indicated that higher SNAP amplitudes reflect an increased number of larger diameter sensory axons with lower depolarization thresholds. Abnormalities in the conduction capacity in axons or receptors are also possible. Patients with non-specific sensory symptoms may have peripheral sensory nerve hyperactivity syndrome, correlating with SNAP high amplitude in the electrophysiological study [24]. Our patients did not complain about obvious sensory disturbances or important pain. In the electrophysiological study we found only one young man with SMA type 3b who had no sensory response.

Our results are generally contrary to the results of other authors, e.g., Sultan et al. [25] who indicated the loss of the SNAP amplitude in patients with SMA types 1 and 2. Similar data were achieved in patients with SMA type 1 by Duman et al. [11]. In their study, 26.7% patients had decreased SNAP amplitude or sensory nerve conduction velocities; in five patients SNAP could not be found. In the first study, statistically lower amplitudes were seen in the median nerve in patients with SMA type 1, while the amplitude did not differ significantly in patients with type 2. In a study by Duman et al. [11] two out of 15 patients with SMA type 1 showed a lack of sensory responses in the median and sural nerves. In the remaining patients, the amplitudes of sensory responses in the median nerve were within normal limits. Most published studies [11, 25–27] on this topic mainly indicate damage to the sural nerve, and therefore this is the one more likely to suffer secondary, additional damage. Pro et al. [26] stressed that SNAP amplitudes in the sural and median nerves are normal in younger patients with SMA type 1, while an axonal neuropathy appears only in older ones.

Our findings suggest sensory fiber overactivity, which seems to confirm previous studies. The predominance of lesions in patients with more advanced disease may reflect a greater intensity of metabolic overactivity as an adaptive mechanism in response to damage. However, a direct comparison of the results obtained between acute forms of SMA in children and chronic forms in adults does not seem valid.

We cannot overlook possible influences of the central nervous system, e.g., the thalamus or cerebellum, which are increasingly considered important in sensory processes, and probably involved in the pathological mechanism in SMA [8–10, 28]. In this context, the analysis of the results obtained in the QST study seems challenging. A QST study is based on the determination of temperature and temperature-induced pain thresholds, which allows assessment of the function of smaller myelinated and unmyelinated sensory fibers – Aδ and C [19, 29]. Thimm et al. [30] revealed significant subclinical small nerve fiber damage in the cornea in patients with SMA type 3, which correlated with motor function. In our QST study, the most significant differences were related to cold pain threshold. SMA patients, especially with milder forms, were significantly less sensitive to pain caused by cold. The threshold for low temperature was comparable in the study groups. The CP-CS difference was greater in SMA patients with less advanced disease but did not reach statistical significance, in contrast to the spread of values for high temperature. HP-WS was significantly greater in the SMA group as a whole, between the individual groups of SMA, and in SMA type 3b compared to the control group. The correlations suggest that in the early stages of the disease, the small fibers responsible for the conduction of temperature and temperature-depended pain show a moderate degree of damage, higher tolerance to more extreme temperatures (low), but with correct sensation thresholds for normal temperatures. CP thresholds appear to be more individualized, varying by body area, depending on complex psychophysical processes. Lötsch et al. [31] demonstrated the hypothesis that CP thresholds reflect the contribution of the two different cold sensors. Fewer heat receptors, greater spatial summation, and more diffuse sensation of heat may underlie the different sensations of pain caused by heat and cold [31–33].

Pitarch-Castellano et al. [34], Sagerer et al. [35], and Uchio et al. [36] described the occurrence of pain in SMA patients. They found a prevalence of pain in 27% to approximately 40% of patients. The pain was of moderate to low intensity, chronic in nature, and predominantly in the lower limbs. The condition of our patients with regard to pain perception was similar to the results mentioned above. Again, it seems that higher pain sensory thresholds in SMA patients may be an adaptive mechanism, active especially in more benign SMA forms. It cannot be ruled out that a deficiency of the systemic SMN protein may cause slight damage to the C and A-delta small fibers early in the course of the disease, which may be evidenced by an increase in pain thresholds.

Vibratory limits were comparable in the patient and control groups. They were significantly higher in the patients with HFSME scores above 10 points. They tended to be higher in SMA type 3b, but this was without statistical significance. These results indicate damage to large A-beta fibers in the prolonged milder form of SMA. Gregory et al. [37] indicated that in amyotrophic lateral sclerosis, vibration thresholds were elevated in comparison to controls. The authors found no reports on vibration sensation in SMA, its possible changes, and significance in the overall view of this disease entity.

In our SMA patients, we were able to show some the features of sensory nerve demyelination. They had significantly lower sensory conduction values with prolonged latencies. These parameters were worse in milder forms of SMA, mainly in type 3b (Fig. 1B) There were no significant differences in sensory conduction velocity or prolongation of latency when comparing patients with more and less disability on the HFSME scale. Therefore, it appears that the demyelination exponents shown in our patients are of relatively low severity, most likely secondary in nature, and this may be dependent on the duration of the disease.

These two findings—early damage to large A-beta fibers, and the secondary demyelination process of sensory fibers—are in agreement with our previous research results on motor fibers based on the conduction velocity distribution study. We found the presence of a coexisting, demyelinating process in the early stage of the disease [14], which was in line with previous reports, e.g., Duman et al. [11].

As a limitation of this study, we must point to the limited number of patients and the fact that the neurophysiological study was restricted to one nerve, as was explained at the beginning of the Discussion section. We must also note the difference in mean age between SMA patients and controls. However, in both groups the mean age range was in the fourth/fifth decade of life, and according to the literature, the important trend for an increase in the results of sensory latency and a decrease in the sensory conduction velocity is observed at ages ≥ 46 years [38]. We also included SMA patients with different comorbidities in the study, and this can potentially promote nerve damage. Among them were underweight and overweight patients, BMI showed differences between the study and control groups and between SMA type 2 and 3 patients. These patients were not excluded due to the low severity of the coexisting diseases, and the lack of clinical and electrophysiological signs of polyneuropathy.

## Conclusions

In conclusion, the high amplitude of sensory potentials on standard neurographic examination suggests sensory fiber hyperactivity, and may be an adaptive mechanism in response to SMN protein deficiency, as well as higher pain sensory thresholds. Damage to small C- and A-delta fibers cannot be excluded in generalized SMN protein deficiency, especially since a concomitant, mild demyelinating process have been found in the early stages of the disease.

## Data Availability

All data may be made available only upon reasonable request to the correspondence author due to the need to protect the privacy of study participants. Correspondence author’s e-mail address: magdalena.koszewicz@umw.edu.pl.

## Abbreviations

BMI: Body mass index
CP: Cold pain
CS: Cold sensation
HFMSE: The Hammersmith Functional Motor Scale-Expanded
HP: Heat pain
M: Median
m/s: Meters per second
ms: Milliseconds
N: Number of cases
QST: Quantitative sensory testing
SD: Standard deviation
SMA: Spinal muscular atrophy
SMN: Survival motor neuron
SNAP: Sensory nerve action potentials
uV: Microvolts
VAS: Visual Analogue Scale
VS: Vibration sensation
WS: Warm sensation
X: Mean
